# Progress of Surgery

**Published:** 1900-10-20

**Authors:** 


### Progress of Surgery.

### SURGERY OF THE VERMIFORM APPENDIX.

**Appendicitis.**—Cottier[1] has examined the ultimate results in 86 cases of appendicitis treated by Broca, and shows that the latter is justified in taking a position against immediate operation in every case, and against searching for an appendix which is at the bottom of a purulent cavity. Of these 86 cases (*a*) 19 went out without operation: of the 19, 5 returned after a fresh attack, and were operated on in the afebrile period; 13 had no pain after the operation and remained well, 1 had slight colicky pains. (*b*) 47 were operated on during the acute attack; 1 for general peritonitis. The appendix was removed from only 3. Of these 47, 28 had no trouble afterwards, and remained in perfect health; 4 went out with a fistula and were subsequently operated upon, and remained in perfect health. (*c*) 30 were operated upon during an interval (8 of them had been operated upon during an acute attack). At the time of writing all 30 were in perfect health, 3 showed slight separation of the muscular walls and a slight impulse on coughing. (*d*) 3 died of intestinal obstruction, at intervals of eight months, one month, and seven months after operation. O'Conor[2] advocates operation as soon as possible after the diagnosis is made, and gives the following reasons:—(1) One cannot say at the outset of any case that spontaneous cure will take place. (2) One is unable to guarantee that perforation without previous formation of protective adhesions may not suddenly occur, which complication is, in the author's experience, invariably fatal. (3) Cœliotomy with removal of the appendix is the most accurate method of diagnosis and cure, and the patient is comforted with the assurance that he will never again suffer from appendicitis. Cox[3] says there is not the slightest doubt that in competent hands the mortality after the removal of the diseased appendix in suitable cases during the quiescent period is practically nothing, and that in acute cases of various kinds (abscess, perforation, &c.) the figure is much lower than any medical form of treatment can possibly show, and will become still lower in proportion to the recognition which timely surgery receives as the only proper way of handling the disease. Baldwin[4] has practised inversion of the appendix in considerably more than a hundred cases. The appendix is freed from adhesions, if present, and brought up into view. The tip of the appendix is held by an assistant with one hand, while the colon is supported just below the origin of the appendix with the other. The meso-appendix is tied off, the ends of the ligature being left long. With scissors the meso-appendix is severed just beyond the ligature, and the tissues constituting it are then seized and stripped off from the appendix from the base to the tip. In some cases the coats are so infiltrated that inversion is possible. In such cases a longitudinal incision is made through the peritoneal and muscular coats, which are then peeled off, leaving merely the mucous membrane intact, which is so thin and soft as to offer little obstacle to inversion. The appendix having thus been prepared for inversion, the tip is seized between the thumb and forefinger and inverted by pressing upon it with the blunt end of a patent-eyed needle. The tip having been inverted for about the length of the needle, the needle is removed and an ordinary long probe substituted. With this the inversion is completed in an instant. Inversion now being complete, one end of the ligature which had been previously used for tying the meso-appendix is threaded into a needle and a single stitch taken across the opening in the bowel which marks the point of disappearance of the inverted appendix. M'Clure[5] quotes Coley on the value of McBurney's point in diagnosis. He says Pepper and Bryant declared that the McBurney point is largely without value, uncertain in its location, apt to be mistaken for points of tenderness due to wholly different causes; and Fowler reports three cases, from eleven operated upon, in which the pain was more marked on the left side, especially at the outer border of the rectus, and the appendix was found to the left of the rectus. Hinder[6] says there is a fair amount of proof that perforation does not take place before twenty-four hours. If then a sharp dose of aperient is given, and operative interference is recommended, provided relief is not experienced within twenty-four or at the outside thirty hours, all danger would be forestalled. If the patient is seen later when a mass is present, it is usually wisest not to be in too great a hurry, for a good zone of adhesions has been formed, and it is impossible for anyone to say in some cases whether pus is present or not. If pus is present, the surgeon's experience of the associated signs and symptoms will, within a short time, settle the diagnosis. There is no doubt but that simply opening the abscess and leaving the appendix alone is the safest method of treatment. Only two or three per cent. subsequently give rise to any trouble. Richardson[7] says—in answer to the question "Should every case be operated upon as soon as the diagnosis of appendicitis is made?"—as a rule the appendix should be removed if the diagnosis is made in the first hours of the attack. After the early hours operation is advisable (1) if the symptoms are severe, and especially if they are increasing in severity; (2) if the symptoms, after a marked improvement, recur; (3) if the symptoms, though moderate, do not improve. The wisdom of the operation is questionable (1) in severe cases in which an extensive peritonitis is successfully localised and the patient is improving; (2) in cases which are at a critical stage, and which cannot successfully undergo the slightest shock. He says—in answer to the question "Should the appendix be removed in every case?"—it should not be removed (1) in localised abscesses with firm walls; (2) when the patient's strength does not permit prolonged search. It should be removed whenever the peritoneal cavity is opened, unless the patient's condition forbids. The appendix should be removed in all cases as soon as the inflammatory process has had time completely to subside—in from two to three months after the attack. In cases simply drained, the scar tissue should be excised, the appendix removed, and the wound securely sutured. He quotes the following conclusion of Greenough on the relation of leucocytosis to appendicitis:—(1) Leucocytosis may be considered a fairly constant symptom of appendicitis; (2) the presence or absence of leucocytosis, or the degree of leucocytosis, without other data, is not sufficient to determine the local condition of the appendix and its surroundings; (3) in a series of cases the degree of leucocytosis corresponds roughly with the degree of temperature, but in individual cases great variations are found; (4) the degree of leucocytosis, when considered in connection with the duration of the attack, is of considerable assistance in the diagnosis of the local condition; (5) a high leucocytosis (above 20,000) on the first or second day of the disease suggests general peritonitis; (6) a low blood-count (below 10,000) after the first week, if accompanied by severe symptoms, indicates general peritonitis, and is of grave prognostic significance, but if accompanied by mild symptoms denotes a mild catarrhal process or well walled-off abscess which has become subacute in character; (7) a high leucocytosis (above 20,000) after the first week or ten days may be taken to indicate a local abscess. Wiener[8] formulates the following conclusions:—(1) Not every case of appendicitis should be operated upon; (2) after a first mild attack, try regulation of diet and salines; (3) after a first severe attack, remove the appendix; (4) after two or more even mild attacks, operate; (5) in an acute attack do not give opium or morphine, and operate during the attack, (*a*) if a chill manifests itself; (*b*) if the pain is severe enough to require morphine; (*c*) if the pulse is very small, or rapid, or irregular; (*d*) if there is persistent vomiting; (*e*) if there is persistent rigidity of the abdominal wall; (*f*) if an abscess can be felt; (*g*) if the general condition makes it imperative; (*h*) if in doubt. Deaver,[9] writing of appendiceal fistulæ, says the causes are as follows:—(1) Abscess formation, (2) migration of bacillus coli communis, (3) separation of the appendix, (4) the use of too many sutures, (5) the prolonged use of drainage tube, (6) pressure necrosis. He urges the abolition of these factors, which can largely be accomplished by immediate action. The treatment of fistulæ is largely dietetic. They should not be frequently washed out, but left severely alone. He summarises as follows:—(1) Fistula is one of the most important sequelæ of appendicitis; (2) some cases close spontaneously; (3) when they produce marked symptoms they should be operated upon; (4) they should be avoided by early operation; (5) fistulæ are generally curable by dietetics; (6) they do not always occur soon after the operation, but may come late in the convalescence.

[1] Treatment, Jan. 25, 1900, p. 709. [2] Lancet, Feb. 3, 1900, p. 295. [3] Med. Rec., Jan. 20, 1900, p. 89. [4] Columbus Med. Jour., March, 1900, p. 112. [5] Ibid., p. 118. [6] Austral. Med. Gaz., Feb. 20, 1900, p. 70. [7] Amer. Jour. Med. Sci., Dec. 1899, p. 629. [8] Med. Rec., May 19, 1900, p. 838. [9] Ibid., June 9, 1900, p. 1005.

